# The predicted impact of tuberculosis preventive therapy: the importance of disease progression assumptions

**DOI:** 10.1186/s12879-020-05592-5

**Published:** 2020-11-23

**Authors:** Tom Sumner, Richard G. White

**Affiliations:** TB Modelling Group, TB Centre, Centre for Mathematical Modelling of Infectious Diseases, Department of Infectious Disease Epidemiology, London School of Hygiene & Tropical Medicine, London, WC1E 7HT UK

**Keywords:** Tuberculosis, Modelling, Structure, Uncertainty

## Abstract

**Background:**

Following infection with *Mycobacterium tuberculosis* (*M.tb*), individuals may rapidly develop tuberculosis (TB) disease or enter a “latent” infection state with a low risk of progression to disease. Mathematical models use a variety of structures and parameterisations to represent this process. The effect of these different assumptions on the predicted impact of TB interventions has not been assessed.

**Methods:**

We explored how the assumptions made about progression from infection to disease affect the predicted impact of TB preventive therapy. We compared the predictions using three commonly used model structures, and parameters derived from two different data sources.

**Results:**

The predicted impact of preventive therapy depended on both the model structure and parameterisation. At a baseline annual TB incidence of 500/100,000, there was a greater than 2.5-fold difference in the predicted reduction in incidence due to preventive therapy (ranging from 6 to 16%), and the number needed to treat to avert one TB case varied between 67 and 157. The relative importance of structure and parameters depended on baseline TB incidence and assumptions about the efficacy of preventive therapy, with the choice of structure becoming more important at higher incidence.

**Conclusions:**

The assumptions use to represent progression to disease in models are likely to influence the predicted impact of preventive therapy and other TB interventions. Modelling estimates of TB preventive therapy should consider routinely incorporating structural uncertainty, particularly in higher burden settings. Not doing so may lead to inaccurate and over confident conclusions, and sub-optimal evidence for decision making.

## Background

Tuberculosis (TB) is an infectious disease caused by the bacteria *Mycobacterium tuberculosis* (*M.tb*). Following infection with *M.tb* it is commonly stated that 10–15% of individuals will develop disease in their lifetime [1]. The risk of developing TB is known to vary with time since infection, with the highest risk in the first year following infection [2]. Disease may occur soon after infection or many years after initial exposure, either through reactivation of “latent” infection or due to re-infection [3, 4]. The mechanisms that underlie this process are incompletely understood.

Mathematical models are frequently used to predict the impact of TB control strategies and to inform policy making. These models use a variety of assumptions when representing progression from infection with *M.tb* to TB disease. Modellers must specify a model structure, the states in the model and the relationships between them, and the model parameters that determine the flows between these states. TB modelling studies often explore the sensitivity of results to parameters (e.g. Sanchez and Blower [5] and Dowdy and colleagues [6]), however the majority of studies using models to predict the impact of control strategies, while often including detailed features such as age-structure, human immunodeficiency virus (HIV) and TB treatment history, employ a single model structure to describe the progression from infection to disease (e.g. [7–13]). A number of studies have explored the impact of model structure in the context of other infectious diseases, including human papillomavirus [14] and rota virus [15] but the importance of model structure has not been widely studied in the context of TB.

Two recent papers [16, 17] compared the predictions of different models for the progression from infection to disease when used to simulate cohorts of recently infected people. The model predictions were compared to data on the incidence of TB by time since *M.tb* infection from various studies. These analyses showed that there is no single “best” model and that several sets of assumptions are consistent with the data. They also found that the assumptions used in many published modelling studies are not consistent with the observed temporal pattern of disease following exposure to *M.tb*. The consequences of using these different assumptions in transmission models used to simulate TB control strategies remain unclear. In particular, do the different “best” models give consistent results when used to simulate interventions, and what are the implications of the use of inconsistent model assumptions?

In this paper we explore how the assumptions (model structure and parameters) used to represent progression from infection to disease effect the predicted impact of a scale up of TB preventive therapy. Preventive therapy (treatment with one or more anti-tuberculosis drugs, typically for a period of 3 to 6 months) reduces the risk of developing TB disease and is recommended by the World Health Organisation (WHO) for high risk populations including people living with HIV, household contacts of pulmonary TB cases and dialysis and organ transplant patients [18]. Previous modelling [11, 19, 20] has highlighted wider use of preventive therapy as a key component of reaching the WHO global TB targets [21]. As preventive therapy aims to prevent progression from infection to disease, it is important to understand how the assumptions used to represent this process may affect the model results.

## Methods

We constructed a set of simple dynamic transmission models to simulate the impact of increased uptake of TB preventive therapy at different TB incidence. To explore the effect of different assumptions about progression from infection to disease we compared the predictions made using 3 different model structures and parameter estimates derived from 2 different data sets.

### Model structures

Our choice of models was informed by a systematic literature review [17] that identified 12 model structures that have been used to describe progression from infection to disease. A summary of all 12 model structures can be found in the appendix.

Our primary analysis focuses on two structures (models 1 and 2) that have been shown to provide the best fit to the observed incidence of TB by time since *M.tb* infection in cohorts of recently exposed people [16, 17].

We also considered a third model structure (model 3) that has been shown to provide a worse fit to the data [16, 17], but has been used in approximately 50% of published modelling studies [17]. It was included here to explore the implications of using an inappropriate structure on the predicted impact of preventive therapy interventions.

**Fig. 1:**
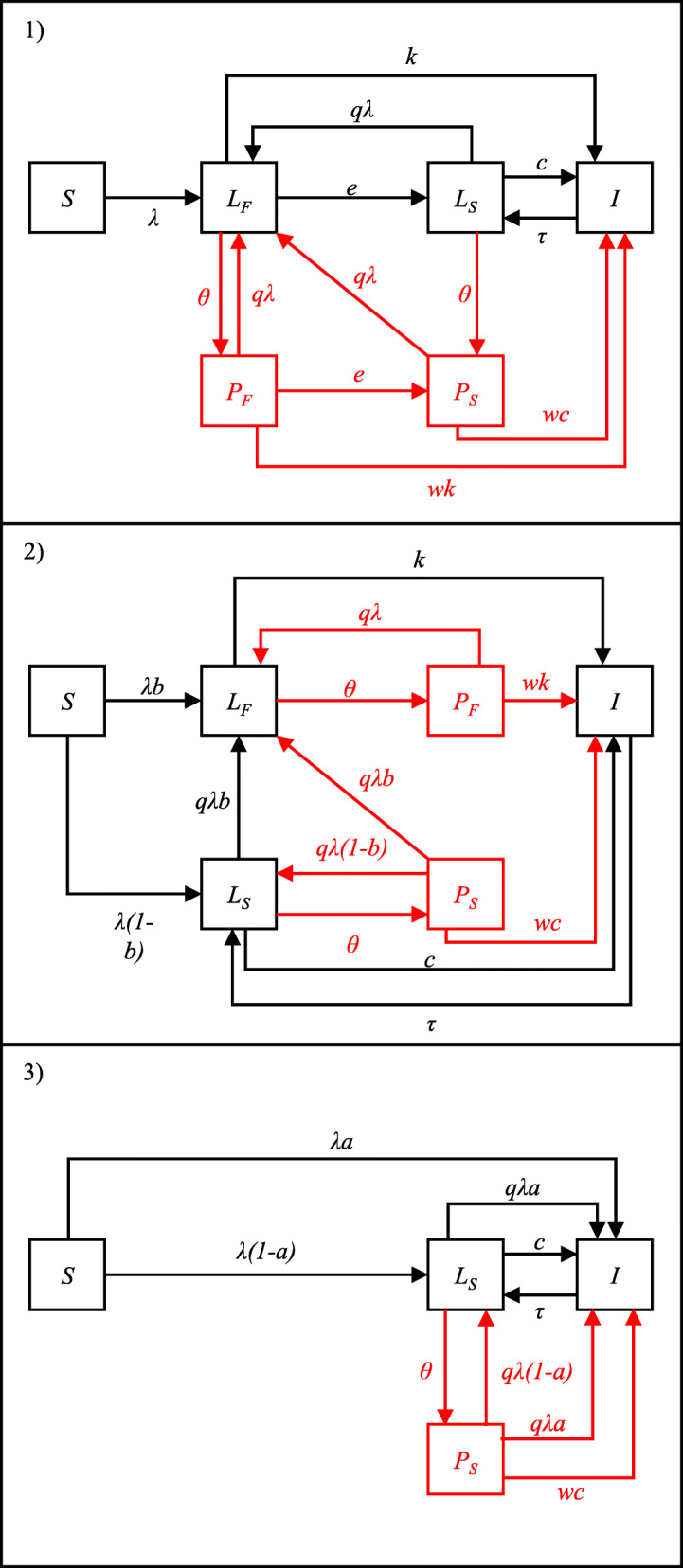
Schematic of model structures. *S* = susceptible; *L*_*F*_ = “fast” latent state; *L*_*S*_ = “slow” latent state; *I* = TB disease; *P*_*F*_ = post preventive therapy (from “fast” latent state); *P*_*S*_ = post preventive therapy (from “slow” latent state). Red lines and boxes show the preventive therapy components of the model. Definitions of model parameters are given in Table 1

Figure 1 shows the different model structures incorporated into a simple dynamic transmission model of TB. The equations and steady state solutions for each of the models are given in the appendix.

The following features are common to all models considered here. Susceptible individuals (*S*) are infected with *M.tb* at a rate *λ* = *βI* where *β* is the rate of effective contact and *I* is the prevalence of TB. Background mortality is modelled at a constant rate, *u*, in all states. In addition, those in the disease state, *I*, are subject to an additional disease associated mortality rate, *m*. The birth rate is set to maintain a constant population size. All births are assumed to be susceptible. In all models we assume that those with TB disease (*I*) are removed back to the “slow” latent state (*L*_*S*_) at a rate, *τ*. This represents effective treatment and natural recovery from disease. Prior exposure to *M.tb*. is assumed to confer some immunity against re-infection. This is captured through the parameter *q* which represents the relative susceptibility to re-infection among those with “slow” latent infection compared to the susceptibility to first infection among previously uninfected individuals.

Model 1 consists of 2 sequential latent states. Following infection all individuals enter the “fast” latent state (*L*_*F*_) where they have an annual rate of progression to disease, *k*. Those who do not develop disease transition to the “slow” latent state (*L*_*S*_), at an annual rate, *e*, where they have an annual rate of disease progression *c* (where *c* < *k*). Biologically, this assumes that all infected individuals have the same risk of developing TB following infection. Individuals in the “slow” latent state (*L*_*S*_) can be re-infected and return to *L*_*F*_.

Model 2 consists of 2 parallel latent states. Following infection, some proportion (*b*) enter the “fast” latent state (*L*_*F*_) where they have an annual rate of progression to disease, *k*. The remainder (1-*b*) enter the “slow” latent state (*L*_*S*_) where they have an annual rate of disease progression *c* (where *c* < *k*). Biologically, this assumes that some proportion of individuals (*b*) are pre-determined to have a high risk of developing TB following infection. Individuals in *L*_*S*_ can be re-infected with a proportion *b* moving to *L*_*F*_ and the remainder remaining in *L*_*S*_.

Model 3 consists of a single “slow” latent state, *L*_*S*_. Following infection, some proportion (*a*) develop disease immediately. The remainder (1-*a*) enter the “slow” latent state where they have an annual rate of disease progression *c*. This can be seen as equivalent to model 2 but with an infinite rate of progression from the fast latent state to disease. Individuals in *L*_*S*_ can be re-infected with a proportion *a* progressing directly to disease and the remainder remaining in *L*_*S*_.

### Model parameterisation

To explore the relative effects of parameter uncertainty compared to structural uncertainty we used parameter estimates for each model structure derived using 2 different data sets. Parameter set A is taken from Menzies et al. [17] and based on data from individuals in the control arms of the British Medical Research Council’s *Bacillus Calmette–Guérin* (BCG) vaccination trials [2] and preventive therapy trials conducted by the United States Public Health Service [22]. Parameter set B is taken from Ragonnet et al. [16] and was obtained by fitting models to data from recent cohorts in the Netherlands [23] and Australia [24]. The parameter values are shown in Table 1. These parameters result in different estimates of the proportion of infected individuals who develop disease over time (see figure A1 in the appendix).

**Table 1:** Parameters for the transmission model

Parameter	Parameter Source	Model		
		1	2	3
***a*,** proportion progressing directly to disease	**A**	–	–	0.0665
	**B**	–	–	0.085*
***b*,** proportion entering fast latent state	**A**	–	0.086	–
	**B**	–	0.09	–
***c*,** rate of progression to disease from slow latent state (per year)	**A**	5.94 × 10^−4^	5.94 × 10^− 4^	3.37 × 10^− 3^
	**B**	2.01 × 10^− 3^	2.01 × 10^− 3^	3.11 × 10^− 3^*
***k*,** rate of progression to disease from fast latent state (per year)	**A**	0.0826	0.955	–
	**B**	0.4015	4.015	–
***e*,** rate of movement from fast latent state to slow latent state (per year)	**A**	0.872	–	–
	**B**	4.015	–	–
***q*,** relative susceptibility to re-infection compared to first infection	0.5 (To explore the role of re-infection in between model differences we also simulated the model with no re-infection, *q* = 0)			
***τ*,** rate of recovery from TB disease (per year)	1 (assumes that the average duration of disease is approximately 1 year)			
***β*,** number of effective contacts (per year)	Varied to produce different TB incidence			
***m*,** rate of TB associated mortality	0.03			
***w,*** relative risk of progressing to disease following preventive therapy	0.4 (In the primary analysis, we assume preventive therapy has an efficacy of 60% against disease progression from prior infection. We explored the impact of varying *w* on the results).			

Parameter set A is taken form Menzies et al. [17], parameter set B is taken from Ragonnet et al. [16]. *These parameters are not reported in Ragonnet et al. [16] so have been estimated by fitting the models to data extracted from figure S14 in [16] – full details are given in the appendix. Parameters in Ragonnet et al. [16] are reported in daily units and have been converted to annual units. “-“indicates the parameter is not used in a given model

For both parameter sets, models 1 and 2 predict the same cumulative incidence over time while model 3 predicts a higher long-term risk of TB than the other models (figure A1). Assuming a life expectancy of 50 years, models 1 and 2 predict a life-time risk of TB of 11% (using parameter set A) and 17% (parameter set B). In contrast model 3 gives a life time risk of 20 and 21% respectively.

### Modelling the impact of preventive therapy

The aim of this analysis was not to make detailed predictions of the impact of preventive therapy, rather to explore how the predicted impact may vary due to the choice of model structure and parameterisation. As such we use a simple model of preventive therapy (shown in red in Fig. 1) similar to that used previously to explore the relationship between TB burden and preventive therapy impact [25].

For each model structure we assumed 5% of the population in all latent states (*θ* = 0.05) is treated with preventive therapy each year. Individuals receiving preventive therapy in the “fast” and “slow” latent states move to corresponding post preventive therapy states, *P*_*F*_ and *P*_*S*_ (note that in model 3 there is only a single latent state and therefore only a single preventive therapy state). We assume that preventive therapy reduces the risk of disease from existing infection but does not prevent against re-infection. This is incorporated in the model via the parameter *w* (see Fig. 1). In our main analysis we assume a reduction of 60% in the risk of progressing to disease [26] (i.e. *w* = 0.4) but also explore the effect of assuming different values for *w*.

We simulated the introduction of preventive therapy into a population in which TB was at an endemic steady state (see appendix for steady state solutions for each model) and explored a range of baseline TB incidence from 0 to 1000/100,000 by varying the number of effective contacts per year (*β*). We calculated the percentage reduction in TB incidence (compared to the endemic equilibrium) after 10 years of preventive therapy. We also calculated the cumulative number of cases averted, the cumulative number of people given preventive therapy and the average number needed to treat (NNT) with preventive therapy to avert one TB case over the 10-year period assuming a constant population of 10,000.

## Results

**Fig. 2:**
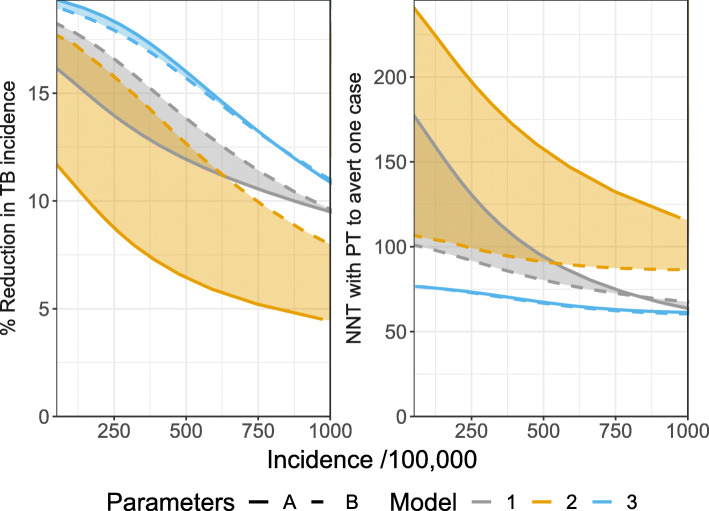
Results of simulating 10 years of preventive therapy as a function of steady state TB incidence. Left: Percentage reduction in TB incidence from steady state equilibrium. Right: average number needed to treat with preventive therapy to avert one case of TB. Colours indicate the different models. Line types indicate the different sources of parameter estimates. Shaded areas illustrate the range of predictions for each model across parameter sets

Figure 2 shows the results of simulating 10 years of preventive therapy for each model structure as a function of the steady state TB incidence assuming 5% annual coverage and a 60% efficacy of preventive therapy against progression to TB disease from prior infection. Colours show the different model structures, solid lines show the results using parameter set A and dashed lines the results using parameter set B.

For all models, the reduction in TB incidence (left panel of Fig. 2) declines as a function of increasing steady state TB incidence; at higher incidence the risk of reinfection after preventive therapy is greater which reduces the long-term benefit of treatment. While the predicted impact declines with increasing incidence, the absolute number of cases averted increases with increasing steady state incidence (not shown) because there are more cases which can be prevented. The number of people treated with preventive therapy also increases with steady state incidence, reflecting the higher prevalence of latent infection, however the NNT (right panel of Fig. 2) is found to decline with increasing incidence. Previous analysis using a model with structure 1 found a non-monotonic relationship between incidence and NNT [25]. In the appendix we show that this behaviour is dependent on the assumed duration of the fast latent state.

There is considerable difference between the predictions using different model structures and parameter sets. For example, at an incidence of 500/100,000, the predicted reduction in incidence (left panel of Fig. 2) ranges from 6 to 16%, a greater than 2.5-fold difference. At the same incidence, the NNT (right panel of Fig. 2) varies from 67 to 157. For each parameter set, model 2 predicts the lowest impact of preventive therapy while model 3 predicts the highest impact. The larger impact predicted by model 3 is due to the fact that this model (as parameterised) results in a higher lifetime risk of developing TB following infection (see figure A1) and therefore the contact rate (β) needed to produce a given TB incidence is smaller (see figure A2). As a result, the risk of reinfection (and therefore TB) after preventive therapy is lower and the predicted impact of the intervention is larger. If model 3 is re-parameterised to give the same lifetime risk of TB as models 1 and 2 we observed a lower impact from model 3. This is because it is not possible to directly prevent fast progression to disease in this model structure by providing preventive therapy to the latent populations; these cases do not pass through a “fast” latent state where they can be treated with preventive therapy (see appendix A5.). Model 2 gives the highest NNT and model 3 the lowest. In the remainder of this section we focus on models 1 and 2.

The effects of assumptions about disease progression are dependent on the TB incidence when preventive therapy is introduced. Table 2 shows the overall range of results compared to the ranges across structures (for a given parameter set) and parameter sets (for a given structure). For a given parameter set, the effect of the model structure on the predictions increases as the incidence of TB increases.

**Table 2:** Predicted percentage reduction in TB incidence

TB Incidence (/100,000)	Reduction in incidence (%)	% of range due to structure		% of range due to parameters	
		Parameters A	Parameters B	Model 1	Model 2
250	8.8–16.6	66%	10%	34%	90%
500	6.5–13.8	74%	16%	26%	84%
750	5.2–11.4	86%	23%	14%	77%
1000	4.5–9.6	98%	32%	2%	68%

Overall range of predicted reduction (due to both model choice and parameters) and the % of the range due to either choice of structure or choice of parameters

**Fig. 3:**
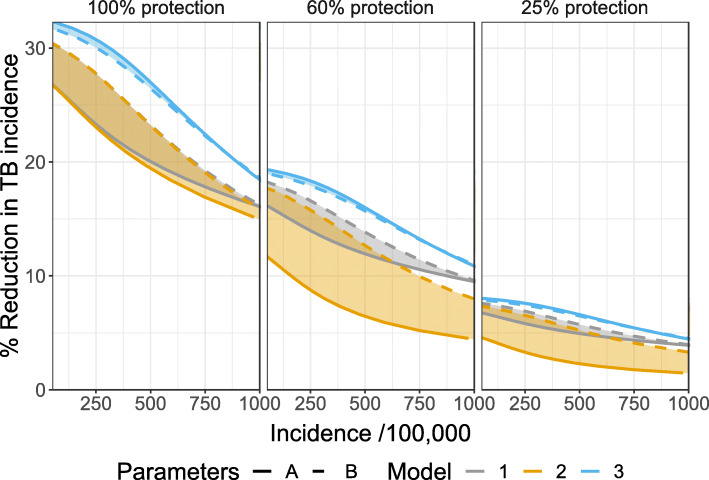
Results of simulating 10 years of preventive therapy as a function of steady state TB incidence for different efficacy of preventive therapy. Colours indicate the different models. Line types indicate the different sources of parameter estimates. Shaded areas illustrate the range of predictions for each model across parameter sets

Figure 3 shows that, as expected, the impact of preventive therapy depends on the assumed efficacy of treatment, but so do the interactions between the assumptions representing disease progression and the model outputs. In particular, the choice between structure 1 or 2 appears to be less influential if the assumed efficacy of preventive therapy is higher.

## Discussion

Our results show that both the model structure and parameter values used to represent progression from infection to disease can affect model predictions of the impact of preventive therapy on TB incidence and the number needed to treat to prevent one case of TB. This highlights the importance of including both structural and parametric uncertainty in TB modelling studies. Failure to do so may result in inaccurate predictions of the potential impact of interventions, and suboptimal evidence for decision making.

Our analysis extends the findings of two previous reviews [16, 17]. Those analyses found that model structures 1 and 2 fit data on the cumulative incidence of TB following infection equally well. In fact, it was shown in Ragonnet et al. [16] that models 1 and 2 produce identical dynamics of TB onset following infection. We show that despite this these models can give markedly different predictions of intervention impact and efficacy. We also find that model 3, which is commonly used but has been found to produce a poor fit to the data, overestimates the impact (and underestimates the NNT) compared to models 1 and 2. This suggests that models using structure 3 could result in inappropriate recommendations for the use of preventive therapy.

We found that the relative importance of the model structure and the choice of parameters depends on the baseline incidence, with the choice of structure becoming more important at higher incidence This is due to the differences in the risk of re-infection between the models which become increasingly important at higher TB incidence: in model 1 all individuals spend some time in the “fast” latent state where they are not at risk of reinfection in contrast to model 2 where only a fraction of individuals pass through the fast latent state. The assumptions around the efficacy of preventive therapy are also important both in determining the overall impact and in the relative effects of model structure and parameterisation.

This work has focussed on the effect of assumptions about progression from infection to disease on the impact of preventive therapy. These assumptions may also affect the predicted impact of other interventions. Other structural assumptions not considered here may also be important. Two previous studies have explicitly considered the role of structural choices in TB modelling. As part of a review of TB modelling Colijn et al. [27] explored how assumptions around the mechanism of protection conferred by prior immunity may affect model predictions. Fojo et al. [28] compared the impact of a hypothetical case finding intervention using three model structures. They found that a model with a single latent state (our model 3) predicted a lower impact than models with sequential high and low risk states (our model 1). This is in agreement with our findings that, in certain circumstances, model 3 can produce inconsistent results.

To allow us to explore a number of different assumptions for the progression from infection to latency the rest of the model was kept as simple as possible. These simplifications may affect our findings. We did not consider HIV or drug resistant forms of TB and we used a very simple representation of demography, assuming a constant population size and a constant life expectancy. Previous work [29] has shown the importance of considering realistic age structure in models of TB transmission. The risks of developing disease have also been shown to differ by age [23, 24, 30] and that simplified models that do not include reactivation may be suitable for modelling paediatric TB [16]. We also assumed TB was in equilibrium before the introduction of preventive therapy, but trends in disease may affect the prevalence of infection and the contribution of ongoing transmission and reactivation to TB incidence. These factors are likely to influence the model predictions of intervention impact and may also affect the interaction between structure, parameters and model outputs. The representation of the preventive therapy intervention was also greatly simplified to explore the impact of model structure on the results and the findings should therefore not be interpreted as predictions of the likely impact of preventive therapy in any specific setting.

To ensure both structural and parametric uncertainty can be explored in a systematic way, standardised methods for incorporating model structure are needed. Approaches such as Bayesian model averaging [31] may be of value for combining predictions from different models, but more work is required to increase their use in infectious disease modelling, in particular to address appropriate methods of weighting different models [32, 33].

In addition to incorporating uncertainty in model predictions it is also important to quantify how different sources of input uncertainty contribute to the variability in outputs. Methods to conduct quantitative sensitivity analysis [34] of both model structure and parameters are needed. Approaches based on factorial sampling [35] analysis of variance [36] or use of regression and classification trees [37] could be utilised to quantify the importance of model structure in TB modelling. Such approaches would allow the key drivers of uncertainty to be identified and focus efforts on collecting data which will reduce uncertainty in future model predictions.

## Conclusion

Uncertainty in model structure is often ignored in TB modelling studies. Future studies should aim to compare results using different structures to ensure the uncertainty in model predictions is captured more accurately. When differences exist in the predictions between models these should be communicated to policy makers, either as discrete scenarios or using more formal methods of model averaging. Not doing so may lead to inaccurate and over confident conclusions, and sub-optimal evidence for decision making.

## Supplementary Information

**Additional file 1.**

## Data Availability

All code used in the study are available at: https://github.com/tomsumner/Latency_Model_Comparison_Public

## Abbreviations

M.tb: *Mycobacterium tuberculosis*
TB: Tuberculosis
HIV: Human immunodeficiency virus
WHO: World Health Organisation
BCG: Bacillus Calmette-Guerin
NNT: Number needed to treat
