# Supplementary material for: The predicted impact of tuberculosis preventive therapy: the importance of disease progression assumptions
Source: BMC Infect Dis. 2020 Nov 23;20:880. doi: 10.1186/s12879-020-05592-5 (PMC7684744; doi:10.1186/s12879-020-05592-5)
Supplement: Supplementary file 1 — Additional file 1. [file 12879_2020_5592_MOESM1_ESM.docx]

**Appendix**

**A.1. Selection of model structures**

Table A1 shows the 12 model structures identified in Menzies et al. [17]. A subset of 6 of these (models 1-6 in table A1) were analysed in Ragonnet et al. [16]. In this work we included 3 of these 12 structures.

Initially we selected all models giving a “good” fit to the data (see table A1). This resulted in 8 possible model structures (models 1,2,6,7,8,9,11,12).

Model 6 was excluded as in both previous studies the best estimate of the parameter “a” was zero, such that this structure is equivalent to model 3. We also excluded model 7 as it includes 3 additional parameters but does not improve the model fit. Models 8 and 9 are rarely used in the published literature. We chose not to consider models 11 and 12 as these were implemented as individual based models in Menzies et al. [17] and our focus here is differential equation models.

Model 3 was shown to provide a marginal fit to the data, however it has been used in a large proportion of published modelling studies; approximately 50% according to the literature review in [17]. We included it here to explore the implications of using an “inappropriate” structure.

| **Model** | | **Parameters** | **% of published studies*** | **Menzies et al. RMSE (qualitative fit)^$^** | **Ragonnet et al. Maximum likelihood** | **Comments** |
| --- | --- | --- | --- | --- | --- | --- |
| 1 | 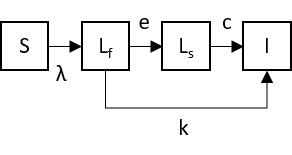 | 3 | 10.6 | 0.0447 (good) | -1254.9 | Included. |
| 2 | 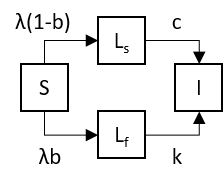 | 3 | 11.2 | 0.0447 (good) | -1254.9 | Included. |
| 3 | 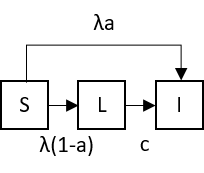 | 2 | 49 | 0.689 (marginal) | -1484.2 | Included for comparison of commonly used “inappropriate” structure. |
| 4 | 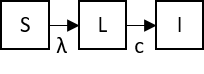 | 1 | 19.2 | 3.07 (poor) | -1484.2 | Excluded as provides poor fit in both studies. |
| 5 | 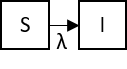 | 0 | 1 | 91.7 (poor) | Not reported | Excluded as provides poor fit in both studies. |
| 6 | 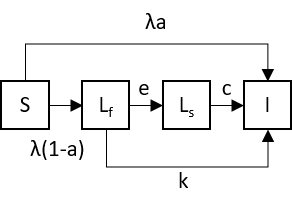 | 4 | 0.3 | 0.0447 (good) | -1254.9 | Excluded. In both studies `a` was estimated to be zero so collapses to model 3. |
| 7 | 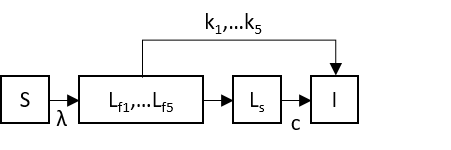 | 6 | 8.7 | 0.0448 (good) | - | Excluded. Includes extra parameters but does not improve fit. |
| 8 | 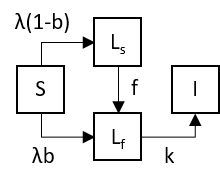 | 3 | 0.6 | 0.0447 (good) | - | Excluded as rarely used in the published literature |
| 9 | 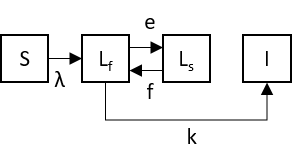 | 3 | 0.6 | 0.0447 (good) | - | Excluded as rarely used in the published literature |
| 10 | 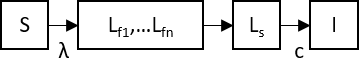 | 5 | 0.3 | 3.09 (poor) | - | Excluded as provides poor fit. |
| 11 | 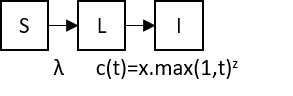 | 2 | 0.3 | 0.0586 (good) | - | Excluded as implemented as individual based model. |
| 12 | 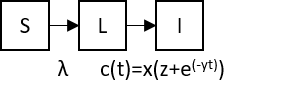 | 3 | 0.3 | 0.0468 (good) | - | Excluded as implemented as individual based model. |

**Table A1.** *% of published studies that used this structure as reported in Menzies et al [17]. ^$^In Menzies et al [17] qualitative fit was defined as poor for RMSE >1, marginal for RMSE between 1 and 0.1 and good for RMSE <0.1.

**A.2. Full details of included models**

Tables A2 and A3. list the variable and parameter names used in the models. Equations and steady state solutions for each of the 3 included models are given below.

| **Variable** | **Description** |
| --- | --- |
| *S* | Susceptible. Never exposed to *M.tb.* |
| *L_F_* | Latently infected with *M.tb*. At risk of “fast” progression to active TB |
| *L_S_* | Latently infected with *M.tb*. At risk of “slow” progression to active TB |
| *I* | Active TB disease |
| *P_F_* | Post-preventive therapy (from *L_F_*) |
| *P_S_* | Post-preventive therapy (from *L_S_*) |
| *T* | Total population |

**Table A2. Model variables**

| **Parameter** | **Description** |
| --- | --- |
| *a* | Proportion progressing directly to disease after infection |
| *b* | Proportion entering fast latent state after infection |
| *c* | Rate of progression to disease from slow latent state (per year) |
| *e* | Rate of movement from fast latent state to slow latent state (per year) |
| *k* | Rate of progression to disease from fast latent state (per year) |
| *m* | Excess mortality rate due to active TB (per year) |
| *q* | Relative susceptibility to reinfection if previously infected |
| *u* | Background mortality rate (per year) |
| *β* | Effective contact rate (per year) |
| *θ* | Rate of starting preventive therapy (per year) |
| *w* | Relative rate of progression to disease from post preventive therapy state (compared to latently infected states). *w* = 0 means preventive therapy completely removes risk of disease from an existing infection. |

**Table A3. Model parameters**

**A.2.1. Model 1**

The model equations are:

$$\begin{aligned} \frac{dS}{dt}=-\left( u+\beta I \right)S+uT+mI\#(A1) \end{aligned}$$

$$\begin{aligned} \frac{{dL}_{F}}{dt}=\beta I\left( S+q\left( L_{S}+P_{F}+P_{S} \right) \right)-\left( k+e+u+\theta\right)L_{F}\#(A2) \end{aligned}$$

$$\begin{aligned} \frac{{dL}_{S}}{dt}=eL_{F}-\left( q\beta I+c+u+\theta\right)L_{S}+\tau I\#(A3) \end{aligned}$$

$$\begin{aligned} \frac{dI}{dt}={kL}_{F}+cL_{S}+w\left( {kP}_{F}+cP_{S} \right)-\left( \tau+u+m \right)I\#(A4) \end{aligned}$$

$$\begin{aligned} \frac{dP_{F}}{dt}=\theta L_{F}-\left( q\beta I+wk+e+u \right)P_{F}\#(A5) \end{aligned}$$

$$\begin{aligned} \frac{dP_{S}}{dt}=\theta L_{S}+eP_{F}-\left( q\beta I+wc+u \right)P_{A}\#(A6) \end{aligned}$$

At steady state, in the absence of preventive therapy (*P_F_*(0) = *P_S_*(0) = *θ* = 0), we have:

$$\begin{aligned} S=\frac{u+mI}{u+\beta I}\#\left( A7 \right) \end{aligned}$$

$$\begin{aligned} L_{F}=\frac{\beta I\left( S+qL_{S} \right)}{k+e+u}\#\left( A8 \right) \end{aligned}$$

$$\begin{aligned} L_{S}=\frac{eL_{F}+\tau I}{q\beta I+c+u}\#\left( A9 \right) \end{aligned}$$

$$\begin{aligned} I=\frac{kL_{F}+cL_{S}}{\tau+u+m}\#\left( A10 \right) \end{aligned}$$

We define $Z=(k+e+u)$ and $Y=(\tau+u+m)$. We first substitute the expression for *L_F_* (equation A8) into the expression for *L_S_* (equation A9) to get:

$$\begin{aligned} L_{S}=\frac{e\beta IS+Z\tau I}{Z\left( q\beta I+c+u \right)-eq\beta I}\#\left( A11 \right) \end{aligned}$$

Then we substitute the expressions for *L_F_* (A8), *L_S_* (A11) and *S* (A7) into the expression for *I* (A10) and multiply through by the denominatiors to obtain an expression in terms of *I* only:

$$\begin{aligned} \left( u+\beta I \right)ZY\left( Z\left( q\beta I+c+u \right)-eq\beta I \right)= \\ k\beta\left( u+mI \right)\left( Z\left( q\beta I+c+u \right)-eq\beta I \right)+\left( kq\beta I+Zc \right)\left( e\beta\left( u+mI \right)+Z\tau\left( u+\beta I \right) \right)\#\left( A12 \right) \end{aligned}$$

Equation A12 can be rearranged to obtain a qudaratic equation for *I* which can be solved to give the steady state value of *I*:

$$\begin{aligned} p_{1}I^{2}+p_{2}I+p_{3}=0\#\left( A13 \right) \end{aligned}$$

Where:

$$\begin{aligned} p_{1}=q\beta^{2}\left( \left( ZY-mk \right)\left( Z-e \right)-k\left( em+\tau Z \right) \right)\#\left( A14 \right) \end{aligned}$$

$$\begin{aligned} p_{2}=\beta Z\left( \left( ZY-mk \right)\left( c+u \right)-c\left( em+\tau Z \right) \right)+u\beta q\left( \left( ZY-\beta k \right)\left( Z-e \right)-k\left( e\beta+Z\tau\right) \right)\#\left( A15 \right) \end{aligned}$$

$$\begin{aligned} p_{3}=uZ\left( \left( ZY-\beta k \right)\left( c+u \right)-c\left( e\beta+Z\tau\right) \right)\#\left( A16 \right) \end{aligned}$$

**A.2.2. Model 2**

The model equations are:

$$\begin{aligned} \frac{dS}{dt}=-\left( u+\beta I \right)S+uT+mI\#\left( A17 \right) \end{aligned}$$

$$\begin{aligned} \frac{dL_{F}}{dt}=b\beta I\left( S+q\left( L_{S}+P_{S} \right) \right)+q\beta IP_{F}-\left( k+u+\theta\right)L_{F}\#\left( A18 \right) \end{aligned}$$

$$\begin{aligned} \frac{{dL}_{S}}{dt}=\left( 1-b \right)\beta I\left( S+qP_{S} \right)-\left( qb\beta I+c+u+\theta\right)L_{S}+\tau I\#\left( A19 \right) \end{aligned}$$

$$\begin{aligned} \frac{dI}{dt}={kL}_{F}+cL_{S}+w\left( kP_{F}+cP_{S} \right)-\left( \tau+u+m \right)I\#\left( A20 \right) \end{aligned}$$

$$\begin{aligned} \frac{dP_{F}}{dt}=\theta L_{F}-\left( wk+q\beta I+u \right)P_{F}\#\left( A21 \right) \end{aligned}$$

$$\begin{aligned} \frac{dP_{S}}{dt}=\theta L_{S}-\left( wc+q\beta I+u \right)P_{S}\#\left( A22 \right) \end{aligned}$$

At steady state, in the absence of preventive therapy (*P_F_*(0) = *P_S_*(0) = *θ* = 0), we have:

$$\begin{aligned} S=\frac{u+mI}{u+\beta I}\#\left( A23 \right) \end{aligned}$$

$$\begin{aligned} L_{F}=\frac{b\beta I\left( S+qL_{S} \right)}{k+u}\#\left( A24 \right) \end{aligned}$$

$$\begin{aligned} L_{S}=\frac{(1-b)\beta IS+\tau I}{qb\beta I+c+u}\#\left( A25 \right) \end{aligned}$$

$$\begin{aligned} I=\frac{kL_{F}+cL_{S}}{\tau+u+m}\#\left( A26 \right) \end{aligned}$$

We define $Y=(\tau+u+m)$. Then we substitute the expressions for *L_F_* (A24), *L_S_* (A25) and *S* (A23) into the expression for *I* (A26) and multiply through by the denominatiors to obtain an expression in terms of *I* only:

$$\left( u+\beta I \right)\left( k+u \right)Y\left( qb\beta I+c+u \right)=$$

$$kb\beta\left( u+mI \right)\left( qb\beta I+c+u \right)+qbk\left( 1-b \right)\beta^{2}I\left( u+mI \right)+qbk\tau\beta I\left( u+\beta I \right)+$$

$$\begin{aligned} \left( k+u \right)c\left( 1-b \right)\beta\left( u+mI \right)+\left( k+u \right)c\tau\left( u+\beta I \right)\#\left( A27 \right) \end{aligned}$$

Equation A27 can be rearranged to obtain a qudaratic equation for *I* which can be solved to give the steady state value of *I*:

$$\begin{aligned} p_{1}I^{2}+p_{2}I+p_{3}=0\#\left( A28 \right) \end{aligned}$$

Where:

$$\begin{aligned} p_{1}=q{b\beta}^{2}\left( u(k+Y) \right)\#\left( A29 \right) \end{aligned}$$

$$\begin{aligned} p_{2}=\left( k+u \right)\beta\left( Y\left( qub+c+u \right)-c\left( \left( 1-b \right)m+\tau\right) \right)-bk\beta(m\left( c+u \right)+qu\left( \beta+\tau\right))\#\left( A30 \right) \end{aligned}$$

$$\begin{aligned} p_{3}=uZ\left( \left( ZY-\beta k \right)\left( c+u \right)-c\left( e\beta+Z\tau\right) \right)\#\left( A31 \right) \end{aligned}$$

**A.2.3. Model 3**

The model equations are:

$$\begin{aligned} \frac{dS}{dt}=-\left( u+\beta I \right)S+uT+mI\#\left( A32 \right) \end{aligned}$$

$$\begin{aligned} \frac{dL_{S}}{dt}=(1-a)\beta I\left( S+qP_{S} \right)-\left( qa\beta I+c+u+\theta\right)L_{S}+\tau I\#\left( A33 \right) \end{aligned}$$

$$\begin{aligned} \frac{dI}{dt}=a\beta I\left( S+q\left( L_{S}+P \right) \right)+c\left( L_{S}+wP_{S} \right)-\left( \tau+u+m \right)I\#\left( A34 \right) \end{aligned}$$

$$\begin{aligned} \frac{dP_{S}}{dt}=\theta L_{S}-\left( q\beta I+u+wc \right)P_{S}\#\left( A35 \right) \end{aligned}$$

At steady state, in the absence of preventive therapy (*P_S_*(0) = *θ* = 0), we have:

$$\begin{aligned} S=\frac{u+mI}{u+\beta I}\#\left( A36 \right) \end{aligned}$$

$$\begin{aligned} L_{S}=\frac{\left( 1-a \right)\beta IS+\tau I}{qa\beta I+c+u}\#\left( A37 \right) \end{aligned}$$

$$\begin{aligned} I=\frac{a\beta I\left( S+qL_{S} \right)+cL_{S}}{\tau+\mu+m}\#\left( A38 \right) \end{aligned}$$

We define$Y=\tau+u+m$. Then we substitute the expressions for *L_S_* (A37) and *S* (A36) into the expression for *I* (A38) and multiply through by the denominatiors to obtain an expression in terms of *I* only:

$$\begin{aligned} Y\left( qa\beta I+c+\mu\right)\left( \mu+\beta I \right)= \\ a\beta\left( \left( u+mI \right)\left( qa\beta I+c+u \right)+q\left( 1-a \right)\beta I\left( u+mI \right)+q\tau I\left( u+\beta I \right) \right)+ \\ c\left( 1-a \right)\beta\left( u+mI \right)+c\tau\left( u+\beta I \right)\#\left( A39 \right) \end{aligned}$$

Equation A39 can be rearranged to obtain a qudaratic equation for *I* which can be solved to give the steady state value of *I*:

$$\begin{aligned} p_{1}I^{2}+p_{2}I+p_{3}=0\#\left( A40 \right) \end{aligned}$$

Where:

$$\begin{aligned} p_{1}=aq\beta^{2}\left( Y-m-\tau\right)\#\left( A41 \right) \end{aligned}$$

$$\begin{aligned} p_{2}=Y\beta\left( qau+c+u \right)-au\beta\left( m+q\left( \beta+\tau\right) \right)-c\beta\left( m+\tau\right)\#\left( A42 \right) \end{aligned}$$

$$\begin{aligned} p_{3}=u^{2}\left( Y-a\beta\right)+cu\left( Y-\beta-\tau\right)\#\left( A43 \right) \end{aligned}$$

**A.3. Cumulative incidence of TB**

Figure A1 shows the cumulative incidence of TB (proportion of cohort experiencing disease) by time since infection by each model structure. Expressions for the final proportion predicted by each model are given below. These results and expressions assume no re-infection occurs.


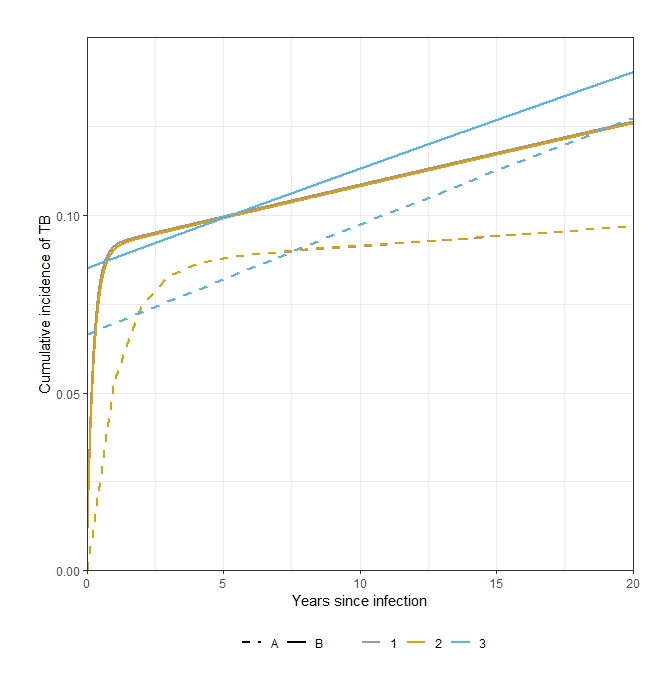


**Figure A1. Cumulative proportion that have developed TB by time since infection.** Colours indicate model, line type indicates source of parameter estimates.

The final proportion of infected individuals who will develop disease in model *i*, *P_i_* is given by:

$$\begin{aligned} P_{1}=\frac{k\left( c+u \right)+ec}{\left( k+e+u \right)\left( c+u \right)}\#\left( A44 \right) \end{aligned}$$

$$\begin{aligned} P_{2}=b\frac{k}{k+u}+\left( 1-b \right)\frac{c}{c+u}\#\left( A45 \right) \end{aligned}$$

$$\begin{aligned} P_{3}=a+\left( 1-a \right)\frac{c}{c+u}\#\left( A46 \right) \end{aligned}$$

Steady state incidence

Figure A2 shows the steady state TB incidence for each model as a function of the contact parameter, *β*.

**
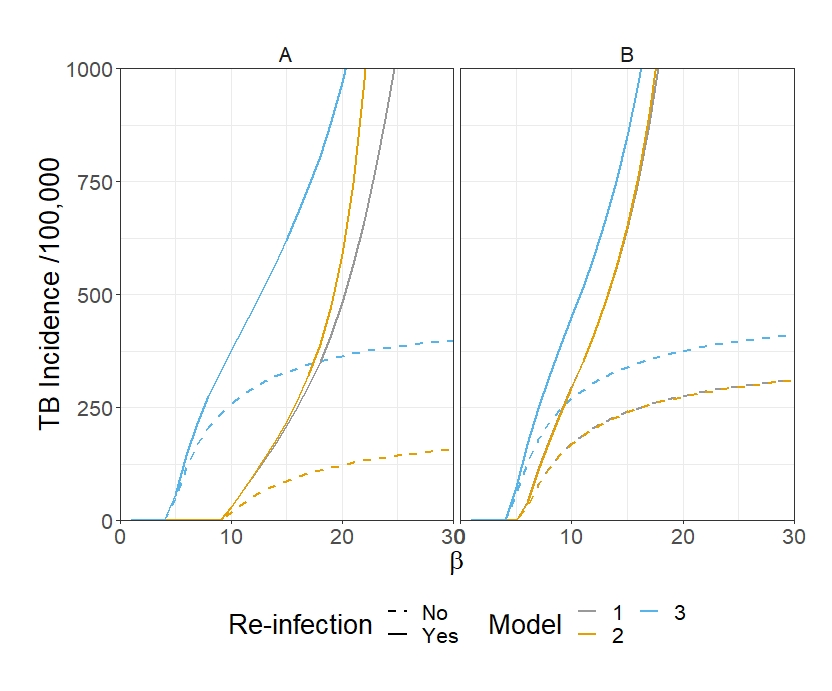
**

**Figure A2. Steady state TB incidence as a function of the contact parameter, *β*.** Left: results using parameter set A; right: results using parameter set B. Colours indicate the different models. Dashed lines show the results when re-infection is not included in the model. Note, results for models 1 and 2 overlap.

In the full model including re-infection (solid lines in figure 3) model 3 results in the highest incidence at a given value of *β* due to the higher lifetime risk of developing TB following infection (see figure A1). At low values of *β* models 1, 2 give the same incidence. However, as the contact rate, *β*, increases the model predictions diverge with a lower incidence predicted by model 1 at a given value of *β*. This divergence occurs due to differences in the risk of reinfection. In model 1 all individuals spend some time in the “fast” latent state where they are not at risk of reinfection and therefore the incidence of disease is lower. This role of reinfection can be seen by contrasting with the results from a model with no reinfection (dashed lines in figure 3). In this case models 1 and 2 predict the same incidence for all values of *β*.

The same qualitative patterns are observed for both parameter sources but the divergence at higher incidence is smaller when using parameter set B.

**A.4. Non-monotonic relationship between NNT and steady state incidence**

Previous analysis of a simple model of preventive therapy [25] found a non-monotonic relationship between baseline incidence and NNT using model structure 1 with a minimum NNT occurring at an incidence in the region of 700/100,000. In this analysis we did not observe this non-monotonic behaviour within the range of incidence explored. The key difference between the parameterisations of model 1 here and the analysis in [25] is the duration of the fast latent state which is determined by *e*, the rate of movement from the fast latent state to the slow latent state. In [25] this was assumed to be 5 years while in the parameterisations used here it is much shorter (between 0.25 and 1.15 years).

To explore this, we compared model 1 with values of *e* of 1, 0.5, 0.3̇33, 0.25 and 0.2 which give durations of the fast latent state of 1 to 5 years. We set the rate of progression from the slow latent state *c* = 5.94x10^-4^ and calculated the value of *k* (the rate of progression from the fast latent state) to give a lifetime cumulative risk of TB of 0.11, the same as with the parameters estimated in Menzies et al. [17].

Figure A3 shows the predicted outputs of the simulated preventive therapy intervention for each duration of the “fast” latent state.

This shows that the non-monotonic behaviour is observed but that the “optimum” incidence (i.e. the value at which the NNT is minimised) is dependent on the duration of the fast latent state. With a duration of 5 years (yellow line) the minimum NNT occurs at an incidence of 1008/100,000. However, with a duration of 1 year (grey line) the minimum NNT occurs at a much higher incidence of 3064/100,000.

**
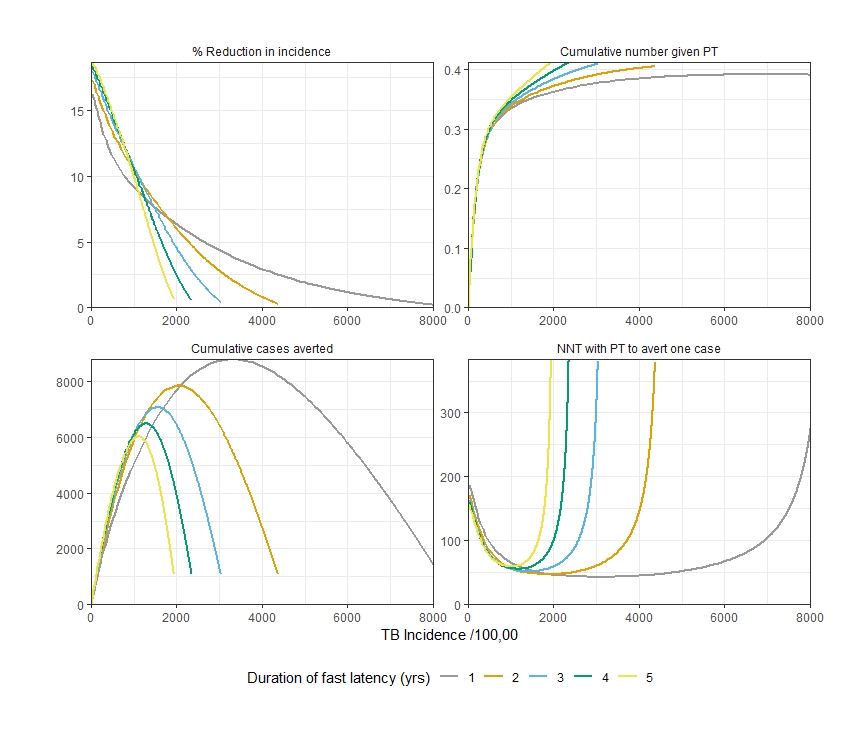
**

**Figure A3. Predicted impact of preventive therapy in model 1 with different durations of “fast” latency.** Top left: Percentage reduction in TB incidence from steady state equilibrium; top right: cumulative number given preventive therapy (assuming a constant population size of 10,000); bottom left: cumulative number of TB cases averted; bottom right: average number needed to treat with preventive therapy to avert one case of TB. Colours indicate the different durations of the “fast” latent state.

**A.5. Re-parameterisation of model 3**

The original parameterisation of model 3 results in an increased lifetime risk of TB compared to models 1 and 2 and a bigger impact of preventive therapy.

Here we explored the effect of re-parameterising model 3 to give the same cumulative probability of TB as models 1 and 2.

Rearranging equation A46 we can obtain an expression for the value of *a*, the proportion progressing directly to disease in model 3 in terms of *c* and the probability of TB (*P_3_*):

$$\begin{aligned} a=\frac{P_{3}-\frac{c}{c+u}}{1-\frac{c}{c+u}}\#\left( A47 \right) \end{aligned}$$

Setting *c* and *P_3_* to the values for models 1 and 2 we can calculate the value of *a* required to give the same cumulative probability of TB in all 3 models. This gives new values for *a* of 0.084 for Menzies and 0.090 for Ragonnet.

Figure A4 shows the predicted impact of the preventive therapy intervention for models 1 and 2 and the original and new parameterisation of model 3.

We find that, in contrast to our main finding the re-parameterised version of model 3 results in a lower predicted impact. This is because it is not possible to directly prevent fast progression to disease in this model structure by providing preventive therapy to the latent populations; these cases do not pass through a “fast” latent state where they can be treated with preventive therapy.


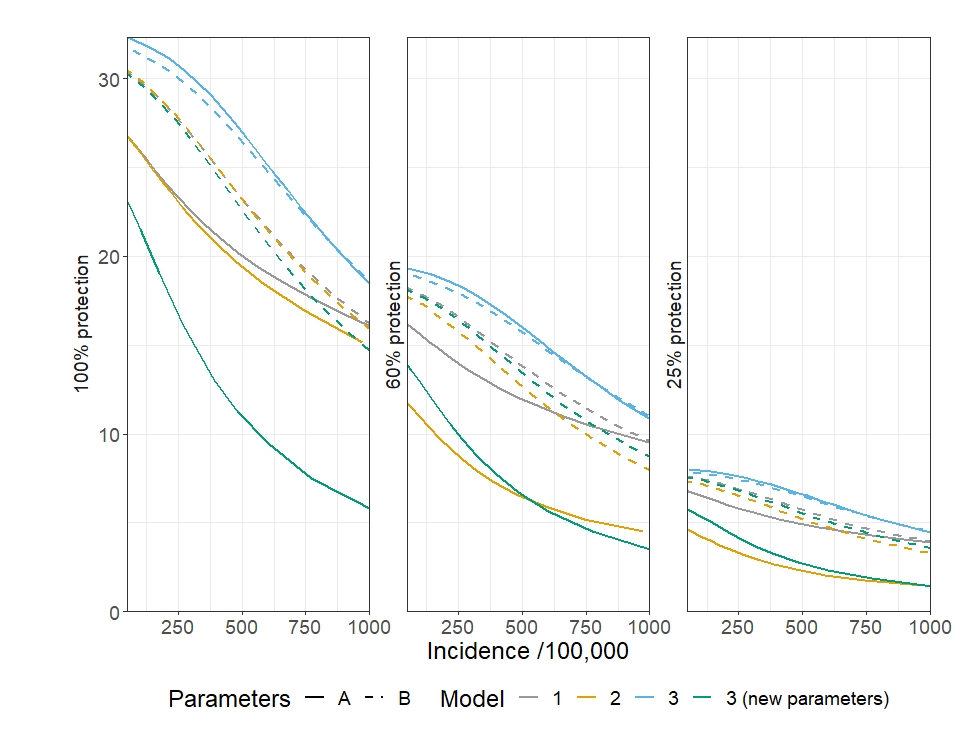


**Figure A4. Predicted impact of preventive therapy with re-parameterisation of model 3.**
